# Tectal plate cyst-associated hydrocephalus in an adult: Case report of a rare clinical entity

**DOI:** 10.1016/j.radcr.2023.10.028

**Published:** 2023-11-04

**Authors:** Marzieh Aalinezhad, Fatemeh Shahnazari, Ali hajihashemi, Mahsa Geravandi

**Affiliations:** a Department of Radiology, Isfahan University of Medical Sciences, Isfahan, Iran; b School of Nursing and Midwifery, Isfahan University of Medical Sciences, Isfahan, Iran

**Keywords:** Tectal plate cyst, Hydrocephalus, Neurologic symptoms

## Abstract

Obstructive hydrocephalus in adults can result from various etiologies, including rare cystic lesions such as tectal plate cysts. To depict a unique case of a tectal plate cyst causing hydrocephalus in an adult accompanied by persistent headaches, visual disturbances, and balance problems. In a clinical context, a 43-year-old female patient presented with a 2-week history of persistent headaches, accompanied by symptoms of dizziness, visual disturbances, and impaired balance. These headaches had exhibited a daily aggravation pattern over a year and were associated with concurrent manifestations of nausea, vomiting, and diplopia. Subsequent neuroimaging through a brain computed tomography (CT) scan disclosed the presence of hydrocephalus. Consultation with a neurologist and brain magnetic resonance imaging (MRI) yielded a diagnosis implicating a tectal plate cyst as the causative agent behind the obstructive hydrocephalus. The patient subsequently underwent surgical excision of the cyst. A follow-up assessment postoperation unveiled a marked improvement in the patient's clinical condition, characterized by the resolution of visual and gait impairments, as well as a notable reduction in the frequency and severity of headaches. This case highlights the importance of considering tectal plate cysts as an uncommon cause of hydrocephalus in the differential diagnosis of patients with persistent headaches and neurological symptoms. Early diagnosis and treatment with surgical removal of the cyst can significantly improve the patient's symptoms and prevent further complications such as hydrocephalus.

## Background

A tectal plate cyst is a rare form of brain cyst that can occur in the tectal plate region [1]. It is important to differentiate tectal plate cysts from other types of cysts that can occur in the quadrigeminal cistern, such as arachnoid cysts [1,2]. Tectal plate cysts generally present as diminutive and asymptomatic entities, detectable via magnetic resonance imaging (MRI) or computed tomography (CT) scans. However, it is noteworthy that in certain cases, they may lead to the development of hydrocephalus, ataxia, and an impaired capacity to perform upward gaze. They have a well-defined border and are round or oblong in shape. Arachnoid cysts are fluid-filled sacs surrounded by a membrane found in the subarachnoid space and differentiate from tectal plate cysts [3]. In the scientific literature, the incidence and prevalence of these cysts are not well established, but they are believed to be uncommon. Tectal plate cysts can occur at any age, but children and adolescents are diagnosed more frequently. Men are marginally more susceptible than women [4].

The etiology of tectal plate cysts remains enigmatic. While they are widely considered to have a congenital origin, the precise underlying mechanism remains elusive. To gain deeper insights into the pathogenesis and prevalence of tectal plate cysts, further investigative research is imperative. The optimal management strategy for tectal plate lesions lacks definitive consensus. While surgical excision may demonstrate efficacy in ameliorating symptoms and averting complications, such as hydrocephalus, alternative therapeutic modalities may exist. Subsequent studies are warranted to undertake comparative analyses and ascertain the most efficacious and least invasive treatment modalities.

## Case presentation

A 43-year-old woman presented to the emergency department with 2 weeks of persistent headaches, accompanied by dizziness, visual disturbances, and balance problems. She had been experiencing intermittent headaches for 1-2 hours daily over the past year, which had become continuous over the last month. These headaches worsened in the morning, gradually improving throughout the day, and were associated with nausea and vomiting. The patient noted that bending and coughing exacerbated the headache. Additionally, she reported double vision and balance issues but did not complain of any memory or concentration deficits. There was no significant medical, surgical, or family history.

Upon admission, the patient exhibited stable hemodynamics, with a blood pressure of 150/100 mm Hg, a pulse rate of 75 bpm, no fever (oral temperature 37°C), and a respiratory rate of 16 breaths per minute. Ophthalmic examination revealed 20/20 visual acuity in both eyes, normal intraocular pressure (12 mm Hg), and an unremarkable anterior eye segment. Fundus examination showed no papilledema.

**Fig. 1:**
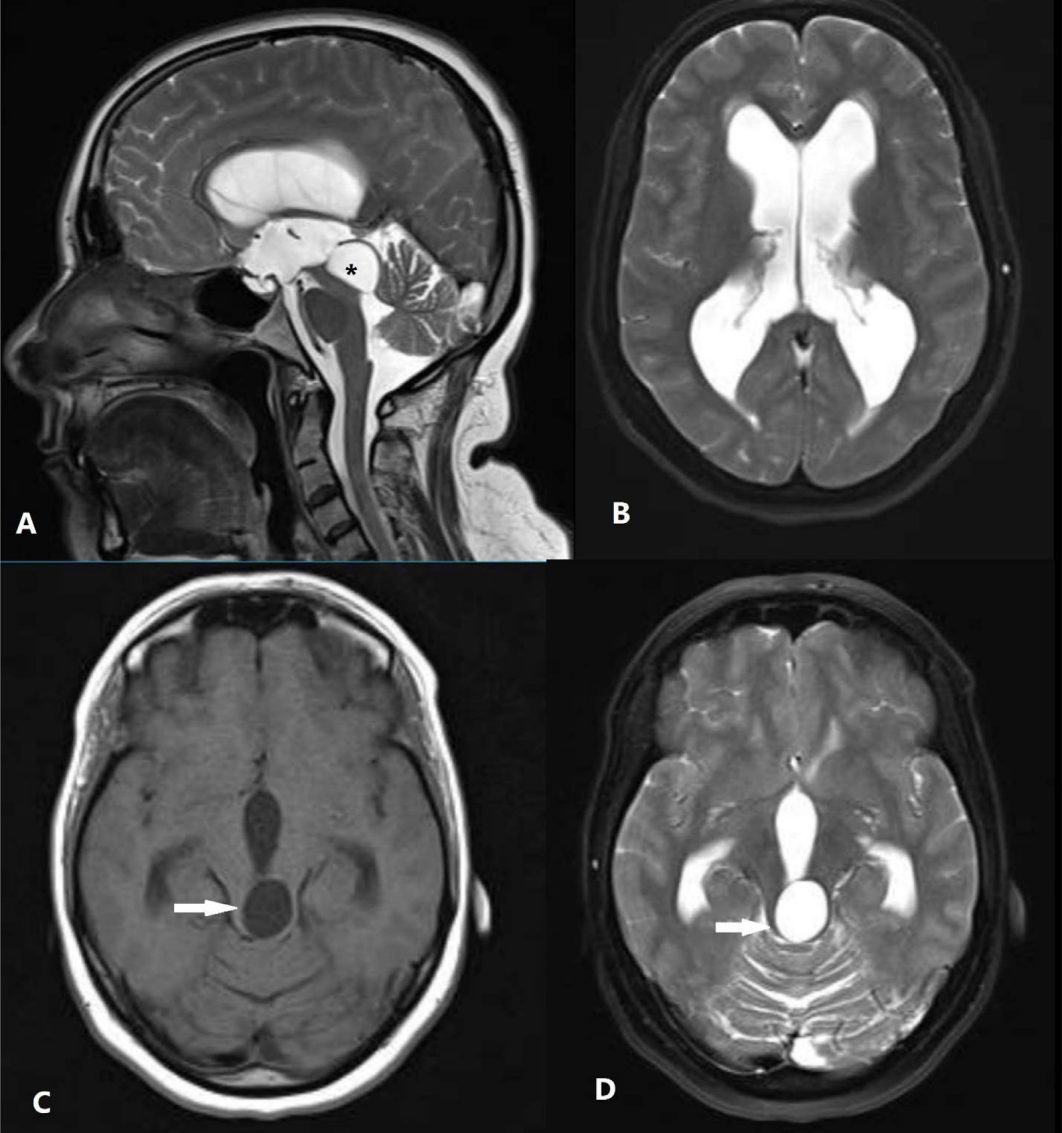
Sagittal and the axial plane of the brain magnetic resonance imaging T2/FLAIR (A, B) sequences: There is a 21*18*20 mm cyst from a tectal plate covered by a thin rim of tectal plate tissue following cerebrospinal fluid (CSF) signal (black asterisk). The axial plane of the brain T1 (C) T2 (D) sequences: The cyst was homogeneously low on T1 and high on T2 sequences (solid white arrows).

Laboratory tests yielded no abnormalities. A brain computed tomography (CT) scan revealed hydrocephalus and cystic lesion in in midbrain. Consultation with a neurologist was recommended. Brain Magnetic resonance imaging (MRI) indicated a 21 × 18 × 20 mm cyst arising from the midbrain, enveloped by a thin rim of tectal plate tissue and exhibiting cerebrospinal fluid (CSF) signal characteristics across all pulse sequences. The cyst exerted pressure on the quadrigeminal cistern and compressed the cerebral aqueduct, resulting in significant obstructive hydrocephalus in the third, fourth, and lateral ventricles of the brain. The cyst appeared homogeneously hypointense on T1 and hyperintense on T2 sequences, with no diffusion restriction on diffusion-weighted imaging (DWI) or evidence of abnormal enhancement (Fig. 1).

Considering these findings, the diagnosis was established as a tectal plate cyst causing obstructive hydrocephalus. Surgical excision of the cyst was performed, leading to a marked improvement in the patient's symptoms. Her recovery was uneventful, and she was discharged 1 week after the procedure. At a follow-up appointment 4 weeks postsurgery, the patient reported substantial symptom amelioration, including the resolution of visual and gait disturbances, as well as reduced headache frequency and severity. Physical examination revealed a steady gait and normal neurological findings. A follow-up brain MRI demonstrated the absence of residual cysts or hydrocephalus.

The patient was advised to maintain close communication with her neurosurgeon and promptly report any recurrence of symptoms. Furthermore, she received guidance to avoid activities that could elevate intracranial pressure, such as strenuous exertion, heavy lifting, and participation in contact sports.

## Discussion

Tectal plate cysts are uncommon developmental anomalies that originate from the tectal plate structure of the midbrain. In the region of the tectal plate, benign cysts are extremely uncommon. By restricting the flow of cerebrospinal fluid (CSF), these cysts can induce obstructive hydrocephalus. The vast majority of tectal plate cysts are asymptomatic and are incidentally discovered during brain imaging investigations. Depending on the size and location of the cysts, when they do produce symptoms, manifestations include hydrocephalus, abnormal gait, urinary incontinence, and deficits in cranial nerves [1].

Upon reviewing the manifestations listed, Hydrocephalus may be the predominant presentation. This particular condition can lead to the development of a variety of uncomfortable symptoms, such as headaches, nausea, vomiting, and visual disturbances. The exact incidence and prevalence of these cysts are not well established in the literature, but they are thought to be uncommon. Tectal plate cysts can occur at any age but are diagnosed more frequently in children and adolescents. They are marginally more prevalent in men than women [4].

In contrast to previous research, we are presenting a case study involving a middle-aged female who has exhibited neurological symptoms. Upon reviewing the literature, we found only 3 publications representing tectal plate cysts. Vandertop et al. reported the case of a 10-year-old boy who presented with headaches and blurred vision. Although the boy did not have double vision or difficulty moving his eyes, a small tectal plate cyst was the cause of his obstructive hydrocephalus [3].

In another study conducted by Kumar et al., 2 cases were reported. One was a 36-year-old male with intermittent headaches for 12-18 months who experienced short-sighted vision and difficulty concentrating at work. He had anisocoria, nystagmus, and eyelid clonus. An MRI showed a cystic lesion from the tectal plate, obstructing the cerebral aqueduct and causing significant lateral and third ventricular enlargement. The other case exhibited a 40-year-old woman with dizzy spells, frequent falls, leg fatigue, and urinary urgency. She had a tonic downward gaze, nystagmus, and normal visual acuity. MRI revealed a tectal plate cyst causing aqueduct stenosis and hydrocephalus. No developmental anomalies were found [1].

The noticeable strength of this study was describing a unique case of a tectal plate cyst causing hydrocephalus in an adult with persistent headaches, visual disturbances, and balance problems. Although the patient underwent surgical intervention and improved significantly, A follow-up assessment of the patient's symptoms confirmed that the cyst had been completely removed. However, our findings should be interpreted by considering the following limitations: We did not have access to the patient's post-operative MRI images, so we relied solely on the report confirming the absence of complications and residual lesions.

## Patient consent

Written informed consent for publication was obtained from the patient.
